# Clinical characteristics and antibody response to Omicron variants among solid carcinoma patients in China on the 2022.12–2023.4 wave of the COVID-19 pandemic

**DOI:** 10.3389/fimmu.2024.1476186

**Published:** 2024-11-06

**Authors:** Rongrong Dai, Weiyu Peng, Nani Xu, Pan Qin, Linling Ding, Qianhui Hua, Jianmin Jiang, Fang He, Hangjie Zhang

**Affiliations:** ^1^ School of Public Health, Hangzhou Medical College, Hangzhou, China; ^2^ Pathogen Microorganism Testing Institute, Shenzhen Center for Disease Control and Prevention, Shenzhen, China; ^3^ Department of Immunization Program, Xihu District Center for Disease Control and Prevention, Hangzhou, China; ^4^ Key Lab of Vaccine, Department of Prevention and Control of Infectious Disease, Zhejiang Provincial Center for Disease Control and Prevention, Hangzhou, China; ^5^ Department of Clinical Laboratory, Zhejiang Provincial People’s Hospital, Hangzhou, Zhejiang, China; ^6^ Department of Prevention and Control of Infectious Disease, Zhejiang Provincial Center for Disease Control and Prevention, Hangzhou, China

**Keywords:** COVID-19, Omicron, solid carcinoma, clinical characteristics, antibody response, long COVID

## Abstract

**Background:**

China experienced a surge of severe acute respiratory syndrome coronavirus 2 (SARS-CoV-2) Omicron variants after adjusting its zero-coronavirus disease 2019 (COVID-19) policy. Although infections with Omicron variants are generally less severe than infections with previous SARS-CoV-2 variants, the clinical characteristics, persistent symptoms, and antibody responses in solid carcinoma patients (SCPs) with COVID-19 during the Omicron wave are unclear.

**Methods:**

We conducted a cross-sectional study in April 2023, recruiting healthy controls (HCs) from the community and SCPs from Zhejiang Provincial People’s Hospital. Serum samples were collected, and a questionnaire was used to assess SARS-CoV-2 infection status, including demographic characteristics, clinical manifestations, and “long COVID” symptoms. Humoral immune responses were analyzed by enzyme-linked immunosorbent assays (ELISAs) targeting immunoglobulin G (IgG) antibodies against the receptor-binding domain (RBD; Omicron BA.4/5) protein and cell culture-based neutralization assays against Omicron variants (BA.4/5, BF.7, XBB.1.5, and EG.5).

**Results:**

In total, 298 SCPs and 258 HCs were enrolled. Self-reported COVID-19 case rates were significantly lower in SCPs than in HCs (78.5% vs. 93.8%, P<0.001). Common COVID-19 symptoms were similar between the two groups, primarily comprising general (92.6% vs. 84.9%) and respiratory symptoms (51.9% vs. 48.2%) after acute infection. There was no significant difference in persistent symptoms at 1–3 months post-infection (P=0.353); fatigue was the most common symptom (45.0% vs. 44.8%). SCPs exhibited lower anti-RBD-IgG titers compared with HCs (1.061 vs. 1.978, P=0.001). The 50% pseudovirus neutralization titer (pVNT_50_) values for prevalent Omicron strains (BA.4/5 and BF.7) were lower in SCPs than in HCs (621.0 [288.8, 1333.0] vs. 894.1 [458.5, 1637.0] and 529.6 [215.3, 1264.5] vs. 463.1 [185.2, 914.0], respectively). Levels of antibodies against subsequent variants (XBB.1.5 and EG.5) also were reduced. There were no significant differences among carcinoma types in the levels of antibodies against Omicron variants. However, SCPs who received the SARS-CoV-2 vaccine or had COVID-19 during the Omicron wave displayed higher antibody levels.

**Conclusions:**

This study elucidated the clinical and immunological characteristics of SCPs during the Omicron wave in China after the shift away from a zero-COVID-19 policy. Our findings provide insights regarding factors that influence COVID-19 symptoms and antibody levels in this population.

## 1 Introduction

The coronavirus disease 2019 (COVID-19) pandemic has been a global health crisis involving widespread economic and social disruption, with over 760 million infections and 6.92 million deaths (1, 2). First detected in South Africa on November 9, 2021, the Omicron variant of severe acute respiratory syndrome coronavirus 2 (SARS-CoV-2) rapidly spread worldwide due to its enhanced transmissibility and immune evasion capabilities (3). At the end of 2022, the Chinese government implemented a dynamic zero-COVID-19 strategy for COVID-19, although all Omicron variants have reduced the pathogenicity, but still a large number of patients infected with BA.5.2 and BF (4). In China, after the policy adjustments for COVID-19, there was a rapid increase in the number of individuals infected with the SARS-CoV-2 (5–7). In the meantime, China’s new ten policies (8) signify a societal reopening during the COVID-19 pandemic, aiming to mitigate its impact on the economy and society.

Among vulnerable populations, solid carcinoma patients have particularly high risks because of their compromised immune systems, which often result from the disease itself or associated treatments. The onset of COVID-19 can further exacerbate their existing conditions (9). Previous studies have shown that, compared with the general population, carcinoma patients exhibit higher risks of severe illness and mortality after contracting SARS-CoV-2 (10–13). A systematic meta-analysis of 14 studies, involving 62,000 COVID-19 patients in various countries, revealed a carcinoma incidence of 6%, significantly higher than the incidence of 0.2% observed in the general population (14). Another meta-analysis of 12,526 Chinese COVID-19 patients showed that carcinoma was a more prevalent comorbidity in those patients than in the general population (15).

Regarding symptoms after COVID-19, a study conducted in Italy revealed that common symptoms included cough, fever, dyspnea, musculoskeletal symptoms (e.g., myalgia, joint pain, and fatigue), gastrointestinal symptoms, and loss of appetite (16). In most cases, vaccinated carcinoma patients with COVID-19 experienced mild symptoms, primarily cough, sputum production, and fever (3, 17). In addition to acute symptoms, “long COVID” has received considerable attention; the reported prevalence of persistent symptoms after contracting COVID-19 ranges from 32.6% to 87% (18). Fatigue and post-exertional malaise are the most common symptoms in individuals with long COVID (19). Notably, antibody stability after contracting COVID-19 appears to be more robust than the stability induced by vaccination (20). Several studies have shown that the vast majority of carcinoma patients develop SARS-CoV-2-specific antibodies after infection, resulting in substantially elevated antibody levels (21). However, more recent findings indicate substantial immune escape for Omicron subvariants, resulting in decreased neutralizing titers associated with mutations in the spike protein (22).

Given the ongoing COVID-19 pandemic and the increased vulnerability of carcinoma patients to severe illness and mortality, it is essential to understand the clinical characteristics of these patients, as well as their immune responses to COVID-19. Specifically, there is a need to investigate differences in immune responses and clinical outcomes between solid carcinoma patients and healthy controls. Accordingly, this study analyzed clinical characteristics, immune responses, and long COVID symptoms in solid carcinoma patients, then compared the findings with data from healthy controls, to provide a comprehensive understanding of the impact of the Omicron wave on this vulnerable population.

## 2 Materials and methods

### 2.1 Study design and participants

**Figure 1:**
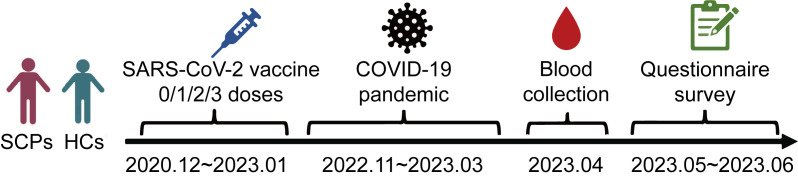
Study design.

This cross-sectional study was conducted in Hangzhou, Zhejiang Province, China, from December 2022 to April 2023, after the wave of the COVID-19 pandemic within China. The study enrolled patients aged 18 and older who had been diagnosed with various types of solid carcinoma at Zhejiang Provincial People’s Hospital; the included carcinoma types were lung, digestive (colorectal, gastric, pancreatic, esophageal, and biliary tract), liver, breast, thyroid, prostate, and other (pituitary, head and neck, ovarian, bladder, renal, ureteral, acoustic neuroma, and uterine). Healthy controls undergoing health examinations at West Lake Community Health Center in Zhejiang Province were selected through sample matching. Blood samples were collected from all participants at both Zhejiang Provincial People’s Hospital and West Lake Community Health Center beginning in April 2023. Most participants had either received 1–3 doses of a COVID-19 vaccine or remained unvaccinated between December 2020 and January 2023 as part of a special population vaccination program. A telephone questionnaire survey was conducted from May to June 2023 to assess the participants’ symptoms after contracting COVID-19. Detailed demographic characteristics and relevant information were also collected from the hospital’s electronic medical record system (**Figure 1**).

### 2.2 Immunological and antibody assessments

In this study, a pseudovirus assay was used to assess the neutralizing antibody potencies of serum samples against SARS-CoV-2 Omicron BA.4/5, BF.7, XBB.1.5, and EG.5 strains of pseudovirus (23). To prepare pseudoviruses, spike proteins with an 18-amino acid deletion from the C-terminus of SARS-CoV-2 PT (Wuhan-1 reference strain, GISAID: EPI_ISL_402119) and the spike protein of each Omicron subvariant were cloned into the pCAGGS vector. Codon optimization was performed to ensure plasmid compatibility with mammalian cell expression, and 30 μg of each construct were transfected into HEK-293T cells. VSV-ΔG-GFP pseudotyped virus was added 24 h after transfection. After 2 h of infection, the VSV-ΔG-GFP residues were removed by changing the medium to fresh complete DMEM medium. After 2 h of infection, the VSV-ΔG-GFP residues were removed by replacing the medium with fresh complete DMEM medium containing anti-VSV-G antibodies (I1-Hybridoma-CRL2700™, ATCC). After incubation for 30 h at 37°C, the supernatants were collected, filtered through a 0.45-μm filter (Cat#SLHP033RB, Millipore), aliquoted, and stored at -80°C. For the pseudovirus neutralization assay, serum samples were initially diluted to 1:8, then the samples were serially diluted two-fold on a 96-well plate. A SARS-CoV-2 pseudotyped virus was added and incubated with the diluted serum for 1 h at 37°C. The mixture was then added to a 96-well plate containing Vero cells. After an additional 20 h of incubation, the number of transducing units (TUs) was determined using the EVOS M7000 Automated Live Cell Fluorescence Imaging System (Thermo Fisher Scientific) in conjunction with ImageJ software. The median serum dilution that neutralized 50% of the virus (pVNT_50_) was calculated by nonlinear regression using GraphPad Prism software. The geometric mean titers (GMTs) of SARS-CoV-2 Omicron variant results are presented as pVNT_50_ titers.

Serum IgG antibodies (from convalescent serum samples) that bind to the SARS-CoV-2 receptor-binding domain (RBD; Omicron BA.4/5) protein were measured using an enzyme-linked immunosorbent assay kit (24). Recombinant RBD (Omicron BA.4/5) protein, identical in amino acid sequence to the target antigen, was coated onto the wells of an enzyme plate. One well served as a blank control (no reagents added), whereas 100 μl of positive and negative controls and diluted samples were added to their respective wells. The plate was sealed and placed in a constant temperature incubator at 37°C for 1 h. If IgG antibodies were present in the sample, they would bind to the pre-coated antigen. Each well was then washed five times with at least 300 μl of washing solution for 30 s each time to remove unbound substances. One hundred microliters of enzyme reagent were added to each well; the plate was then sealed and placed in a constant temperature incubator at 37°C for 30 min. After an additional washing step, a color development solution containing tetramethylbenzidine (TMB) was added to each well, and the plate was placed in a constant temperature incubator at 37°C in the dark for 15 min to allow color development. Finally, 50 μl of stop solution was added to each well to terminate the reaction, and the absorbance (OD_450-630_) values were read at wavelengths of 450 nm and 630 nm. The anti-RBD-IgG (Omicron BA.4/5) titer results are presented as OD_450_-OD_630_.

### 2.3 Statistical analysis

Demographic characteristics and symptoms after SARS-CoV-2 infection were collected for both solid carcinoma patients and healthy controls. Categorical variables, such as sex and age, are presented as numbers (percentages); they were analyzed using Pearson’s Chi-squared test or Fisher’s exact test. Continuous variables, such as pVNT_50_, are expressed as the median (interquartile range) [M (P25, P75)] for non-normally distributed data. Multiple independent groups meeting normality and homogeneity of variance assumptions were compared using one-way analysis of variance (ANOVA). Non-parametric Kruskal–Wallis and Mann–Whitney U tests were used for continuous variables that did not follow a normal distribution. Binary logistic regression and multiple linear regression were utilized for multifactorial analysis. All statistical tests were two-sided, and P-values <0.05 were considered statistically significant. All analyses were conducted using R version 4.3.1 (R Foundation for Statistical Computing) and GraphPad Prism 9 software.

## 3 Results

### 3.1 Demographic characteristics

This study included 298 solid carcinoma patients and 258 healthy controls. The participants’ demographic characteristics are presented in **Table 1**. Among the solid carcinoma patients, there were 138 men (46.3%) and 160 women (53.7%), whereas the healthy controls comprised 114 men (44.2%) and 144 women (55.8%). Regarding age, 117 solid carcinoma patients (39.3%) were aged 18–59 years, and 181 (60.7%) were aged ≥60 years. Among the healthy controls, 76 (29.5%) were aged 18–59 years and 182 (70.5%) were aged ≥60 years. The proportion of participants aged 18–59 years was significantly greater among solid carcinoma patients than among healthy controls (P=0.015). In terms of body mass index (BMI), 66 carcinoma patients (22.1%) and 188 healthy controls (72.9%) had a BMI <25 kg/m^2^; 19 carcinoma patients (6.4%) and 69 healthy controls (26.7%) had a BMI ≥25 kg/m^2^; 213 carcinoma patients (71.5%) and 1 healthy controls (0.4%) had unknown BMI. Regarding COVID-19 vaccination status, 177 solid carcinoma patients (59.4%) had received the vaccine, whereas 121 (40.6%) had not. Among the healthy controls, 256 (99.2%) had received the vaccine and two (0.8%) had not. The proportion of unvaccinated individuals was significantly greater among solid carcinoma patients than among healthy controls (P<0.001). Among the solid carcinoma patients, 22 had lung carcinoma (7.4%), 83 had digestive carcinoma (27.9%), 21 had liver carcinoma (7.0%), 50 had breast carcinoma (16.8%), 21 had thyroid carcinoma (7.0%), 32 had prostate carcinoma (10.7%), and 70 had other carcinomas (23.5%).

**Table 1:** Participant demographic characteristics.

Variable	Solid carcinoma patients (N=298)	Healthy controls (N=258)	*p*
Sex			
Male	138 (46.3)	114 (44.2)	0.616
Female	160 (53.7)	144 (55.8)	
Age, years			
18-59	117 (39.3)	76 (29.5)	0.015
≥60	181 (60.7)	182 (70.5)	
Median (*P_25_, P_75_*)	63 (53,71)	67 (54,73)	
BMI (kg/m^2^)			
<25.0	66 (22.1)	188 (72.9)	0.411
≥25.0	19 (6.4)	69 (26.7)	
unknown	213 (71.5)	1 (0.4)	
Vaccine (s) administered			
Yes	177 (59.4)	256 (99.2)	<0.001
No	121 (40.6)	2 (0.8)	
COVID-19 infection			
Yes	234 (78.5)	242 (93.8)	<0.001
No	64 (21.5)	16 (6.2)	
Type of carcinoma			
Lung carcinoma	22 (7.4)	–	
Digestive carcinoma	83 (27.9)	–	
Liver carcinoma	21 (7.0)	–	
Breast carcinoma	50 (16.8)	–	
Thyroid carcinoma	21 (7.0)	–	
Prostate carcinoma	32 (10.7)	–	
Other carcinomas	70 (23.5)	–	

Data are presented as number of participants (%) or median (*P_25_, P_75_*). ^a^Digestive carcinoma (colorectal carcinoma, gastric carcinoma, pancreatic carcinoma, esophageal carcinoma, biliary tract carcinoma); ^b^Other carcinomas (pituitary carcinoma; carcinomas of the mouth, nose, and throat; ovarian carcinomas; bladder carcinoma; renal carcinoma; ureter carcinoma; acoustic nerve carcinoma; uterine carcinoma). Statistical analyses were conducted using the Chi-squared test.

### 3.2 Analysis of COVID-19 clinical characteristics and associated factors

#### 3.2.1 Clinical characteristics of COVID-19 in solid carcinoma patients and healthy controls

After the 2022.12-2023.4 wave of the COVID-19 pandemic in China, we conducted a questionnaire to assess COVID-19 status among the study participants (**Table 2**). In terms of general symptoms after contracting COVID-19, 50 solid carcinoma patients (92.6%) and 169 healthy controls (84.9%) experienced systemic symptoms; the difference was not statistically significant (P=0.143). Respiratory symptoms were reported by 28 solid carcinoma patients (51.9%) and 96 healthy controls (48.2%); the difference was not statistically significant (P=0.638). Digestive tract symptoms were observed in five solid carcinoma patients (9.3%) and 10 healthy controls; this difference also was not statistically significant (P=0.399). However, 38 solid carcinoma patients (76.0%) and 95 healthy controls (54.6%) experienced fever with a body temperature ≥38°C (P=0.007). Regarding muscle soreness, 26 solid carcinoma patients (48.1%) reported this symptom; although this proportion was greater than that among healthy controls (73, 36.7%), the difference was not statistically significant (P=0.126). Concerning the timing of recovery after contracting COVID-19, nine solid carcinoma patients (16.7%) and 43 healthy controls (22.6%) experienced symptoms for ≥1 week; again, the difference was not statistically significant (P=0.354). In terms of medical visits after contracting COVID-19, 11 solid carcinoma patients (20.4%) and 34 healthy controls (17.8%) sought medical care; this difference was not statistically significant (P=0.667).

**Table 2:** Symptoms of COVID-19.

Variable	SCPs	HCs	P
**General symptom**			0.143
Yes	50(92.6)	169(84.9)	
No	4(7.4)	30(15.1)	
**Respiratory symptom**			0.638
Yes	28(51.9)	96(48.2)	
No	26(48.1)	103(51.8)	
**Digestive tract symptom**			0.399
Yes	5(9.3)	10(5.0)	
No	40(90.7)	189(95.0)	
**Fever**			0.007
<38°C	12(24.0)	79(45.4)	
≥38°C	38(76.0)	95(54.6)	
**Muscular soreness**			0.126
Yes	26(48.1)	73(36.7)	
No	28(51.9)	126(63.3)	
**Symptom recovery time**			0.354
<1 weeks	45(83.3)	148(77.5)	
≥1 weeks	9(16.7)	43(22.5)	
**Medical visits after COVID-19 infection**			0.667
Yes	11(20.4)	34(17.8)	
No	43(79.6)	157(82.2)	
**Time of SARS-CoV-2 antigen or PCR turns to negative**			0.031*
<3 weeks	36(92.3)	143(99.3)	
≥3 weeks	3(7.7)	1(0.7)	

Data are presented as number of participants (%). Statistical analyses were conducted using the Chi-squared test and Fisher’s exact test*.

#### 3.2.2 Factors influencing COVID-19 onset

In the single-factor analysis of solid carcinoma patients, 159 individuals (89.8%) who received the SARS-CoV-2 vaccine and 75 unvaccinated individuals (62.0%) contracted COVID-19; this difference was statistically significant (P<0.01) (**Table 3**). The main independent variables in multifactorial analysis were the presence of COVID-19 and associated symptoms (general, respiratory, digestive, fever, and muscle soreness); the dependent variables were sex, age, body mass index, vaccination status, and malignancy type. The likelihood of contracting COVID-19 was significantly higher among participants who received the SARS-CoV-2 vaccine than among unvaccinated participants (odds ratio (25)=91.5; 95% confidence interval, 2.0–4086.5) (**Supplementary Table 1**).

**Table 3:** Associations between participant characteristics and COVID-19 symptoms in solid carcinoma patients.

Variable	COVID-19 infection		General symptom		Respiratory symptom		Digestive tract symptom		Fever (≥38°C)		Muscular soreness	
	N (%)	p	N (%)	p	N (%)	p	N (%)	p	N (%)	p	N (%)	p
Sex												
Male	112 (81.2)	0.303	20 (95.2)	0.953	8 (38.1)	0.107	2 (9.5)	1.000	15 (78.9)	0.967	9 (42.9)	0.535
Female	122 (76.3)		30 (90.9)		20 (60.6)		3 (9.1)		23 (74.2)		17 (51.5)	
Age, years												
18-59	89 (76.1)	0.407	23 (95.8)	0.771	15 (62.5)	0.161	2 (8.3)	1.000	19 (82.6)	0.313	12 (50.0)	0.808
≥60	145 (80.1)		27 (90.0)		13 (43.3)		3 (10.0)		19 (70.4)		14 (46.7)	
BMI (kg/m2)												
<25.0	59 (89.4)	0.831	41 (95.3)	0.181*	24 (55.8)	0.249	5 (11.6)	0.546	33 (82.5)	0.082	21 (48.8)	0.841
≥25.0	16 (84.2)		9 (81.3)		4 (36.4)		0 (0.0)		5 (50.0)		5 (45.5)	
Vaccine (s) administered												
Yes	159 (89.8)	<0.001	48 (92.3)	1.000*	27 (51.9)	1.000*	5 (9.6)	1.000*	36 (75.0)	1.000*	25 (48.1)	1.000*
No	75 (62.0)		2 (100.0)		1 (50.0)		0 (0.0)		2 (100.0)		1 (50.0)	
Type of carcinoma												
Lung carcinoma	15 (68.2)	0.211	3 (75.0)	0.634*	2 (50.0)	0.518*	0 (0.0)	0.758*	1 (25.0)	0.136*	1 (25.0)	0.297*
Digestive carcinoma	66 (80.5)		9 (100.0)		3 (33.3)		2 (22.2)		7 (77.8)		5 (55.6)	
Liver carcinoma	17 (85.0)		1 (100.0)		1 (100.0)		0 (0.0)		1 (100.0)		1 (100.0)	
Breast carcinoma	34 (68.0)		12 (92.3)		9 (69.2)		2 (15.4)		8 (66.7)		8 (61.5)	
Thyroid carcinoma	17 (81.0)		5 (100.0)		3 (60.0)		0 (0.0)		3 (75.0)		4 (80.0)	
Prostate carcinoma	29 (90.6)		6 (85.7)		2 (28.6)		0 (0.0)		4 (80.0)		2 (28.6)	
Other carcinomas	55 (78.6)		14 (93.3)		8 (53.3)		1 (6.7)		14 (93.3)		5 (33.3)	

Data are presented as number of participants (%). Statistical analyses were conducted using the Chi-squared test and Fisher’s exact test*.

### 3.3 Analysis of immunogenicity

#### 3.3.1 SARS-CoV-2 antibody levels in solid carcinoma patients and healthy controls

**Figure 2:**
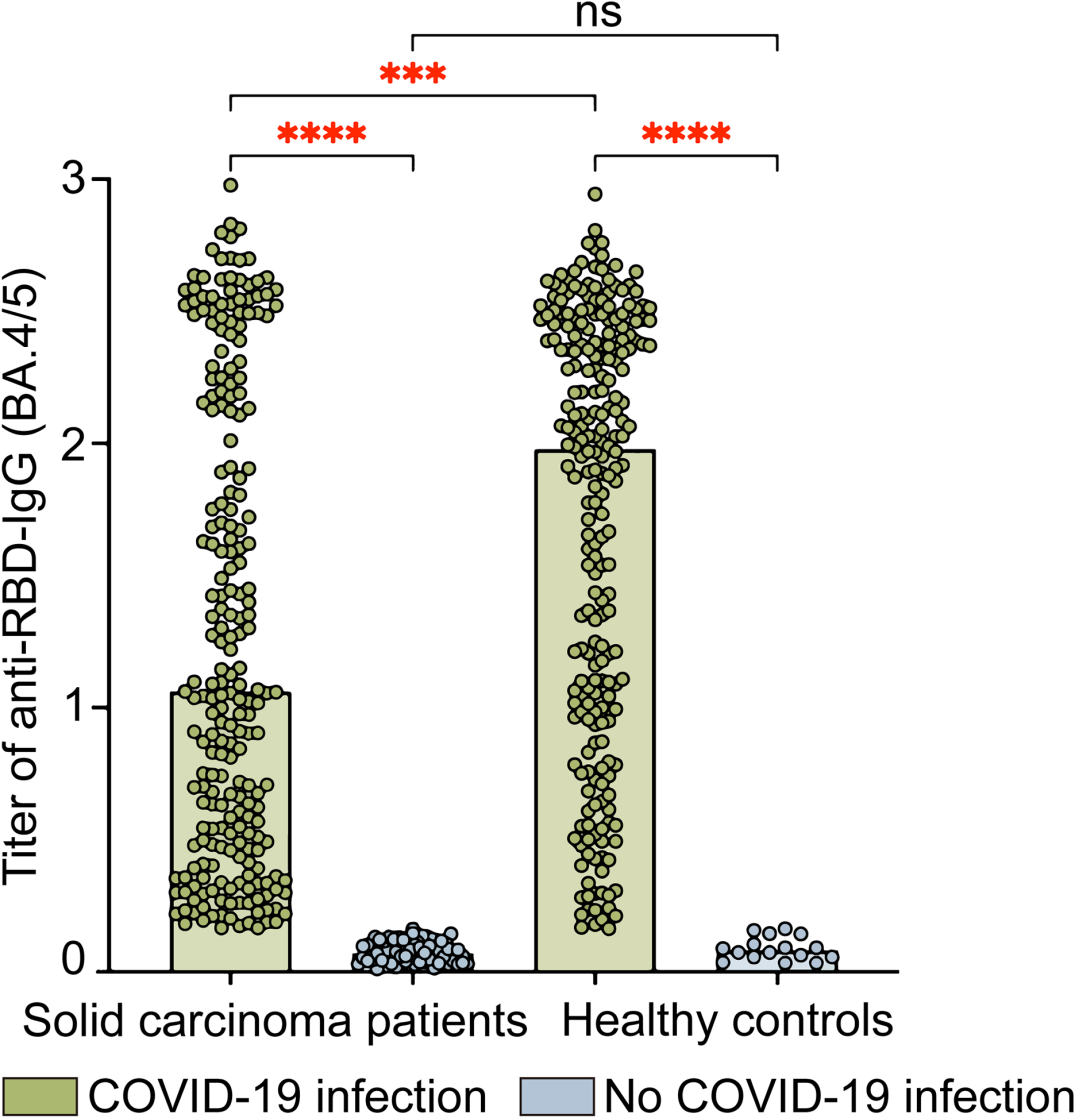
Levels of IgG antibodies against SARS-CoV-2 RBD (Omicron BA.4/5) protein in solid carcinoma patients and healthy controls. This figure compares the anti-RBD-IgG (BA.4/5) titers in SARS-CoV-2 infected and uninfected individuals for both solid carcinoma patients and healthy controls. The anti-RBD-IgG titers are presented as absorbance (OD_450-630_); the values were read at wavelengths of 450 nm and 630 nm. Statistical analyses were conducted using the Kruskal–Wallis test. ns, not significant; *P<0.05; **P<0.01; ***P<0.001; ****P<0.0001.

Regarding IgG antibody levels against the SARS-CoV-2 RBD (Omicron BA.4/5) protein, solid carcinoma patients who contracted COVID-19 had a value of 1.1 (0.5, 2.2), whereas patients who did not contract COVID-19 had a value of 0.1 (0.0, 0.1); this difference was statistically significant (P<0.001). Among healthy controls, the values for those who did and did not contract COVID-19 were 2.0 (1.0, 2.4) and 0.1 (0.1, 0.1), respectively; again, the difference was statistically significant (P<0.001). Among individuals with SARS-CoV-2 infection, neutralizing antibody levels were significantly lower in solid carcinoma patients than in healthy controls (P=0.0001) (**Figure 2**).

**Figure 3:**
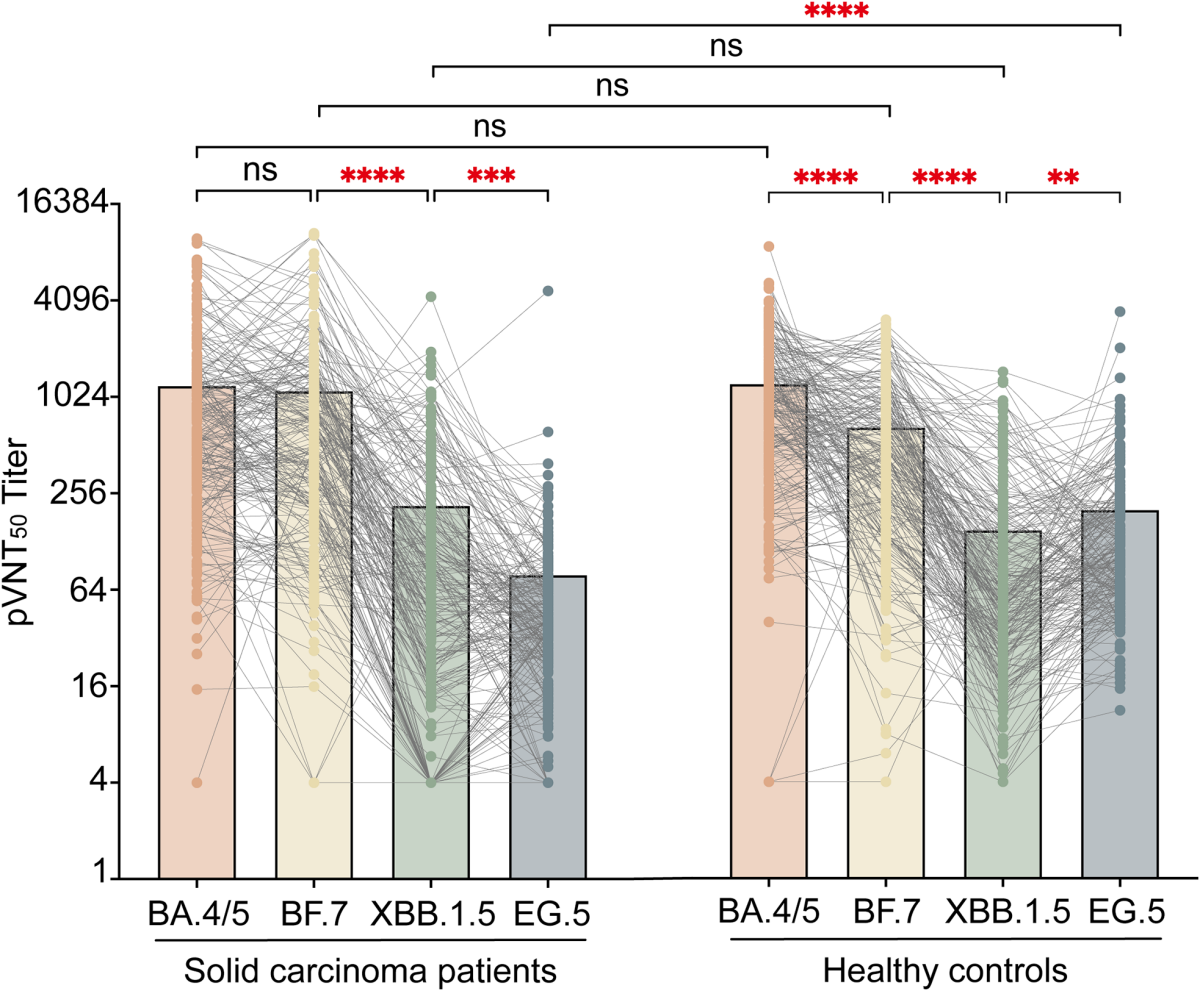
pVNT_50_ against SARS-CoV-2 variants in solid carcinoma patients and healthy controls. This figure presents the pVNT_50_ against Omicron variants (BA.4/5, BF.7, XBB.1.5, and EG.5) in SARS-CoV-2 infected solid carcinoma patients and healthy controls. Statistical analyses were conducted using the Kruskal–Wallis test. ns, not significant; *P<0.05;**P<0.01;***P<0.001; ****P<0.0001.

Neutralization testing against SARS-CoV-2 Omicron subvariants (BA.4/5, BF.7, XBB.1.5, and EG.5) in solid carcinoma patients and healthy controls with COVID-19 was conducted using pseudoviruses (**Figure 3**). Among solid carcinoma patients, the GMTs of pVNT_50_ were 621.0 (288.8, 1333.0), 529.6 (215.3, 1264.5), 66.1 (17.8, 201.1), and 38.6 (21.7, 67.8) for BA.4/5, BF.7, XBB.1.5, and EG.5, respectively. Statistically significant differences were observed between BA.4/5 and XBB.1.5/EG.5 (P<0.001), BF.7 and XBB.1.5/EG.5 (P<0.001), and XBB.1.5 and EG.5 (P=0.0006). Among healthy controls, the GMTs of pVNT_50_ were 894.1 (458.5, 1637.0), 463.1 (185.2, 914.0), 59.3 (29.0, 163.6), and 106.8 (61.2, 217.3) for BA.4/5, BF.7, XBB.1.5, and EG.5, respectively. Significant differences were evident between BA.4/5 and XBB.1.5/EG.5 (P<0.001), BF.7 and XBB.1.5/EG.5 (P<0.001), and XBB.1.5 and EG.5 (P=0.0015). Among all individuals with COVID-19, solid carcinoma patients and healthy controls showed a significant difference in the GMT of pVNT_50_ for EG.5 (P<0.001).

**Figure 4:**
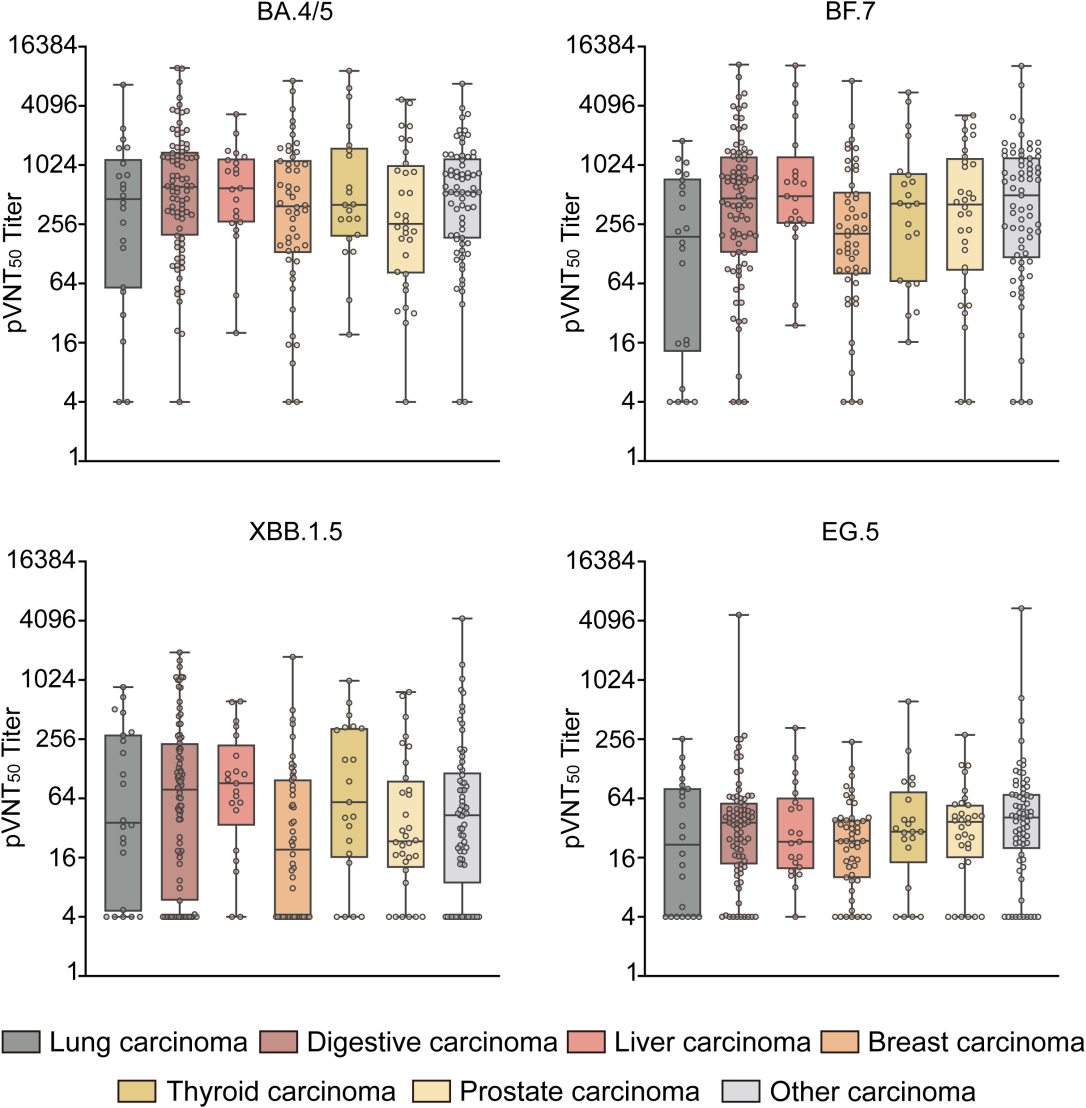
pVNT_50_ against Omicron variants in patients with different types of solid carcinoma. This figure illustrates the pVNT_50_ against Omicron variants (BA.4/5, BF.7, XBB.1.5, and EG.5) in patients with various types of solid carcinoma (lung, digestive, liver, breast, thyroid, prostate, and other). Statistical analyses were conducted using the Kruskal–Wallis test.

The GMTs of pVNT_50_ against Omicron BA.4/5, BF.7, XBB.1.5, and EG.5 variants were tested in healthy controls and patients with different types of solid carcinoma (e.g., lung, digestive, liver, breast, thyroid, prostate, and other) (**Figure 4**). Regarding BA.4/5 antibody levels, digestive carcinoma showed the highest pVNT_50_ (614.6 [196.0, 1398.0]), followed by liver carcinoma (593.9 [266.7, 1194.5]); prostate carcinoma displayed the lowest pVNT_50_ (258.1 [80.9, 1023.8]). Concerning BF.7 antibody levels, other carcinomas showed the highest pVNT_50_ (503.9 [115.7, 1223.0]), followed by liver carcinoma (492.7 [258.3, 1254.6]); lung carcinoma displayed the lowest pVNT_50_ (191.3 [12.9, 747.2]). In terms of XBB.1.5 antibody levels, liver carcinoma showed the highest pVNT_50_ (91.2 [33.7, 226.5]), followed by digestive carcinoma (78.7 [5.8, 233.0]); breast carcinoma displayed the lowest pVNT_50_ (19.3 [4.0, 99.8]). With respect to EG.5 antibody levels, other carcinomas showed the highest pVNT_50_ (41.0 [19.6, 71.1]), followed by prostate carcinoma (37.0 [15.8, 55.3]); lung carcinoma displayed the lowest pVNT_50_ (21.7 [4.0, 81.6]) (**Figure 4**).

#### 3.3.2 Factors influencing SARS-CoV-2 antibody levels

In the single-factor analysis of IgG antibody levels against the SARS-CoV-2 RBD (Omicron BA.4/5) protein in solid carcinoma patients, levels were significantly higher among vaccinated individuals (1.3 [0.5, 2.3]) than among unvaccinated individuals (0.3 [0.1, 0.7]) (P<0.001). Similarly, BA.4/5 antibody levels were significantly higher among vaccinated patients (708.9 [263.5, 1413.0]) than among unvaccinated patients (295.1 [97.8, 780.1]) (P<0.001). BF.7 antibody levels also were significantly higher among vaccinated patients (431.7 [151.9, 1010.5]) than among unvaccinated patients (273.9 [53.2, 1052.2]) (P=0.042). Furthermore, solid carcinoma patients who contracted COVID-19 had significantly higher IgG antibody levels against the SARS-CoV-2 RBD (Omicron BA.4/5) protein (1.1 [0.5, 2.2]) compared with the levels in patients who did not contract COVID-19 (0.1 [0.0, 0.1]) (P<0.001). BA.4/5 antibody levels were significantly higher among patients with COVID-19 (621.0 [288.8, 1333.0]) than among patients who did not develop COVID-19 (122.8 [33.7, 329.9]) (P<0.001). Similarly, BF.7 antibody levels were significantly higher among patients with COVID-19 (529.6 [215.3, 1264.5]) than among patients who did not develop COVID-19 (40.2 [11.0, 134.4]) (P<0.001) (**Table 4**).

**Table 4:** Associations between participant characteristics and antibody levels in solid carcinoma patients.

Variable	Anti-RBD IgG		BA.4/5		BF.7	
	Titer	p	GMT	p	GMT	p
Sex						
Male	0.9 (0.2,2.1)	0.079	569.9 (206.1,1387.0)	0.138	516.8 (137.0,1259.8)	0.013
Female	0.6 (0.2,1.6)		444.3 (146.9,1210.0)		297.2 (87.5,836.5)	
Age, years						
18-59	0.7 (0.2,1.6)	0.195	420.9 (163.9,1207.0)	0.634	312.6 (76.5,920.7)	0.161
≥60	0.8 (0.2,1.9)		538.1 (174.9,1248.8)		404.8 (125.0,1096.0)	
BMI (kg/m2)						
<25.0	1.3 (0.5,2.4)	0.906	627.5 (255.7,1405.3)	0.756	294.7 (112.5,923.4)	0.681
≥25.0	1.6 (0.5,2.2)		833.7 (178.3,1243.0)		622.1 (84.0,1100.0)	
Vaccine (s) administered						
Yes	1.3 (0.5,2.3)	<0.001	708.9 (263.5,1412.5)	<0.001	431. (151.9,1010.5)	0.042
No	0.3 (0.1,0.7)		295.1 (97.8,780.1)		273.9 (53.2,1052.2)	
COVID-19 infection						
Yes	1.1 (0.5,2.2)	<0.001	621.0 (288.8,1333.0)	<0.001	529.6 (215.3,1264.5)	<0.001
No	0.1 (0.0,0.1)		122.8 (33.7,329.9)		40.2 (11.0,134.4)	
Type of carcinoma						
Lung carcinoma	1.0 (0.1,2.4)	0.734	461.4 (56.9,1177.3)	0.496	191.3 (12.9,747.2)	0.041
Digestive carcinoma	0.8 (0.3,1.6)		614.6 (196.0,1398.0)		467.2 (131.4,1250.0)	
Liver carcinoma	0.7 (0.2,1.3)		593.9 (266.7,1194.5)		492.7 (258.3,1254.6)	
Breast carcinoma	0.6 (0.1,1.4)		390.6 (130.8,1149.8)		205.1 (79.2,548.6)	
Thyroid carcinoma	0.7 (0.3,1.6)		402.8 (191.4,1536.5)		415.5 (66.2,845.9)	
Prostate carcinoma	0.8 (0.3,2.1)		258.1 (80.9,1023.8)		406.8 (86.3,1208.0)	
Other carcinomas	1.1 (0.2,2.2)		542.2 (183.0,1197.5)		503.9 (115.7,1223.0)	

Data are presented as median (P_25_, P_75_). Statistical analyses were conducted using the non-parametric Mann–Whitney test.

In the multifactorial analysis, the main independent variables were IgG antibody levels against the SARS-CoV-2 RBD (Omicron BA.4/5) protein, BA.4/5 antibody levels, and BF.7 antibody levels. The dependent variables included sex, age, body mass index, vaccination status, and malignancy type. Compared with unvaccinated participants, vaccinated participants had higher IgG antibody levels against the SARS-CoV-2 RBD (Omicron BA.4/5) protein (B=1.4; 95% confidence interval, 0.2–2.5) (**Supplementary Table 2**).

### 3.4 Symptoms of long COVID

The proportions of participants with long COVID symptoms were 37.0% (20 individuals) among solid carcinoma patients and 30.4% (58 individuals) among healthy controls; the difference was not statistically significant (P=0.353). Among solid carcinoma patients, the most common symptom of long COVID symptom was fatigue (45.0%, nine cases), followed by dyspnea (20.0%, four cases). Among healthy controls, fatigue also was the most common symptom of long COVID (44.8%, 26 cases), followed by throat discomfort (15.5%, nine cases) (**Table 5**).

**Table 5:** Symptoms of long COVID.

Variable	Solid carcinoma patients	Healthy controls	p
**Persistent symptoms**			0.353
Yes	20(37.0)	58(30.4)	
No	34(63.0)	133(69.6)	
Symptoms			
Fatigue	9(45.0)	26(44.8)	0.989
Sleepy	3(15.0)	6(10.3)	0.574
Headache/dizziness/migraine	1(5.0)	4(6.9)	1.000
Cough	2(10.0)	7(12.1)	1.000
Discomfort in the pharynx	1(5.0)	9(15.5)	0.409
Taste/smell change or loss	3(15.0)	6(10.3)	0.876
Gastrointestinal discomfort	1(5.0)	0(0.0)	0.256*
Breath/shortness of breath	4(20.0)	4(6.9)	0.216
Decreased exercise capacity	2(10.0)	2(3.5)	0.577
Arrhythmia/palpitations	0(0.0)	3(5.2)	0.565*
Chest pain	0(0.0)	1(1.7)	1.000*
Sleep disorder	1(5.0)	2(3.4)	1.000*
Tinnitus/earache	0(0.0)	2(3.4)	1.000*

Data are presented as number of participants (%). Statistical analyses were conducted using the Chi-squared test and Fisher’s exact test*.

## 4 Discussion

As the COVID-19 pandemic persists, the virus continues to rapidly evolve, leading to the emergence of several Omicron variants that have become dominant worldwide, hitherto these types of mutant strains have caused extensive breakthrough infections both in China and around the world (26, 27). Some of these variants exhibit the ability to evade immunity conferred by prior infections or vaccinations, posing a renewed threat to public health (28–30).

In the present study, the proportion of vaccinated solid carcinoma patients was lower than that of healthy controls. This discrepancy may be attributed to various factors influencing vaccine hesitancy and uptake among carcinoma patients. These findings are consistent with the results of previous studies, which have frequently linked vaccine hesitancy in this population to concerns about vaccine-related side effects, uncertainty about vaccine efficacy and safety, and ongoing aggressive cancer treatments (31, 32). Intriguingly, we observed a higher rate of COVID-19 cases among vaccinated solid carcinoma patients than among unvaccinated patients. An online questionnaire in China revealed that COVID-19 vaccination may exacerbate some upper respiratory symptoms, such as sore throat, nasal congestion, and runny nose (33). This phenomenon may have occurred because vaccinated solid carcinoma patients engaged in more social activities and had increased opportunities for exposure, whereas unvaccinated individuals were more cautious about potential infection.

Regarding symptoms of COVID-19, solid carcinoma patients were more likely than healthy controls to experience fever with a maximum temperature of ≥38°C, suggesting that fever is more severe in carcinoma patients. However, the symptoms observed in this study were manageable. Multiple studies have linked SARS-CoV-2 infection to cough, sputum production, fever, and dyspnea in carcinoma patients (3, 34). Another study focusing on children and young adults with cancer revealed that all participants experienced mild to moderate symptoms after contracting COVID-19 (35). Additionally, the proportion of solid carcinoma patients who required ≥3 weeks to achieve negative results in a SARS-CoV-2 antigen or PCR was higher than the corresponding proportion of healthy controls, suggesting a longer recovery time in carcinoma patients.

Regarding the immune response to the Omicron variant of SARS-CoV-2, individuals with COVID-19 generally exhibit higher immunogenicity than those who do not contract COVID-19. However, solid carcinoma patients show lower antibody levels compared with healthy controls. A study by Donze et al. revealed that antibody levels, particularly anti-SARS-CoV-2 IgG antibodies targeting the S1 domain of the Spike protein, were significantly elevated among carcinoma patients with COVID-19 (35). Other studies have shown that antibody levels remain stable for 12 months after the initial infection (36, 37). In our study, vaccinated solid carcinoma patients with COVID-19 demonstrated stronger immunogenicity than their unvaccinated counterparts. Across different Omicron strains, both BA.4/5 and BF.7 elicited higher antibody levels than XBB.1.5 or EG.5 in both solid carcinoma patients and healthy controls. A study conducted in Beijing showed that neutralizing antibody levels against the prototypic (PT), BA.2, BA.4/5, and BF.7 strains were significantly elevated among individuals with COVID-19. Conversely, the subvariants BM.1.1.1, CH.1.1, XBB, XBB.1.5, and XBB.1.16 showed the lowest neutralizing antibody titers (38). Concerning the BF.7 strain tested in our study, male solid carcinoma patients exhibited stronger immunogenicity than female patients. Interestingly, our study revealed significant differences in the immune response to XBB.1.5 and EG.5 between solid carcinoma patients and healthy controls. Data from a study in South Korea showed lower levels of antibodies to the EG.5 variant than to the XBB.1.5 variant, similar to results in our solid carcinoma patients (39). In addition, another study pointed out that EG.5 is more infectious than XBB.1.5 in some areas, and 20% of the infection rate in the United States is related to EG.5, which may cause a part of the population to be more susceptible to SARS-CoV-2 EG.5 variant, resulting in higher antibody levels after infection EG.5 than XBB.1.5. This is consistent with what we have observed in healthy controls (40). At the same time, these differences may also be related to immunosuppression, prior immune response, and the effect of immunotherapy in solid carcinoma patients. Among different types of solid carcinomas, patients with other carcinomas displayed the highest BF.7 antibody levels, followed by liver carcinoma; patients with lung carcinoma exhibited the lowest levels. In terms of long COVID symptoms, the incidences did not significantly differ between solid carcinoma patients and healthy controls; fatigue was the most common symptom in both groups. A study by Soriano et al. showed that 10%–30% of individuals with COVID-19 experience persistent symptoms, including fatigue, cognitive impairment, shortness of breath, and mental disorders (41). A meta-analysis of 81 studies revealed that the proportion of individuals experiencing fatigue at ≥12 weeks after COVID-19 diagnosis was 0.32, whereas the proportion with cognitive impairment was 0.22 (42).

Our study had some limitations. First, the sample size was small, limiting the generalizability of the findings. Additionally, there was a low number of patients with each type of solid carcinoma. Second, because this was a cross-sectional survey, we could not confirm Omicron infection status through laboratory testing. Third, some areas of data collection were incomplete; we lacked key clinical records, such as patient treatment methods and specific dates of tumor diagnosis. These gaps in data may have introduced bias in our analysis of the impact of SARS-CoV-2 on solid carcinoma patients. Additionally, our research conclusions are drawn from the Omicron wave in China, and thus may not be fully generalizable to other countries with different healthcare systems or COVID-19 policies, and the variant studied is Omicron, which suggests that the findings might not apply to other variants of concern.

In conclusion, we found that solid carcinoma patients exhibited hesitancy and concerns regarding COVID-19 vaccination. They were more likely to develop fever after contracting COVID-19 and tended to exhibit slower recovery times. Both solid carcinoma patients and healthy controls demonstrated increased immunogenicity after contracting COVID-19, although solid carcinoma patients displayed lower immunogenicity compared with healthy controls. Vaccinated individuals in both groups showed higher immunogenicity after contracting COVID-19, compared with unvaccinated individuals. Antibody levels varied across Omicron strains; the levels were highest for antibodies against BA.4/5 and lowest for antibodies against EG.5. Antibody levels against Omicron BF.7 also varied according to solid carcinoma type; the levels were highest in patients with other carcinomas and lowest in patients with lung carcinoma. Additionally, we found that male solid carcinoma patients had higher antibody levels against Omicron BF.7. These findings suggest that factors such as sex, disease severity, and treatment regimens influence the immune responses of solid carcinoma patients to vaccines and viruses. Further research is needed to fully understand the impacts of these differences on clinical outcomes.

## Data Availability

The original contributions presented in the study are included in the article/**Supplementary Material**. Further inquiries can be directed to the corresponding authors.

## References

[1] Xie D Choi HK Dalbeth N Wallace ZS Sparks JA Lu N. Gout and excess risk of severe SARS-coV-2 infection among vaccinated individuals: A general population study. Arthritis Rheumatol. (2023) 75:122–32. doi: 10.1002/art.42339 PMC9537980 36082457

[2] Ruhl L Kühne JF Beushausen K Keil J Christoph S Sauer J. Third SARS-coV-2 vaccination and breakthrough infections enhance humoral and cellular immunity against variants of concern. Front Immunol. (2023) 14:1120010. doi: 10.3389/fimmu.2023.1120010 37033958 PMC10073596

[3] Liu L Liao Y Yu X Rong L Chen B Chen G. Clinical characteristics and prognostic factors of COVID-19 infection among cancer patients during the december 2022 - february 2023 omicron variant outbreak. Front In Med. (2024) 11:1401439. doi: 10.3389/fmed.2024.1401439 PMC11171418 38873204

[4] Huang S Gao Z Wang S. China's COVID-19 reopening measures-warriors and weapons. Lancet. (2023) 401:643–4. doi: 10.1016/S0140-6736(23)00213-1 PMC9949835 36841613

[5] Zhang W Wang R Jin P Yu X Wang W Zhang Y. Clinical Characteristics and Outcomes of Liver Transplant Recipients Infected by Omicron During the Opening up of the Dynamic Zero-Coronavirus Disease Policy in China: A prospective, Observational Study. Am J Transplant. (2024) 24:631–40. doi: 10.1016/j.ajt.2023.09.022 37863433

[6] Who COVID-19 Dashboard . Available online at: https://Data.Who.Int/Dashboards/COVID-19/Cases?M49=156 (Accessed October 15th, 2024).

[7] Chen Y Lin Y Lu H Wu X Pan Y Xia A. Real-World Effectiveness of Molnupiravir, Azvudine and Paxlovid against Mortality and Viral Clearance among Hospitalized Patients with COVID-19 Infection During the Omicron Wave in China: A Retrospective Cohort Study. Diagn Microbiol Infect Dis. (2024) 109:116353. doi: 10.1016/j.diagmicrobio.2024.116353 38776665

[8] Zero-COVID Policy Keeps Pandemic under Control . Available online at: https://english.www.gov.cn/news/topnews/202201/10/content_WS61db8e5cc6d09c94e48a3610.html (Accessed October 15th, 2024).

[9] Wekking D Senevirathne TH Pearce JL Aiello M Scartozzi M Lambertini M. The impact of COVID-19 on cancer patients. Cytokine Growth Factor Rev. (2024) 75:110–8. doi: 10.1016/j.cytogfr.2023.11.004 38103990

[10] Song Q Bates B Shao YR Hsu FC Liu F Madhira V. Risk and outcome of breakthrough COVID-19 infections in vaccinated patients with cancer: real-world evidence from the national COVID cohort collaborative. J Clin Oncol. (2022) 40:1414–27. doi: 10.1200/jco.21.02419 PMC9061155 35286152

[11] Song NJ Chakravarthy KB Jeon H Bolyard C Reynolds K Weller KP. Mrna vaccines against SARS-coV-2 induce divergent antigen-specific T-cell responses in patients with lung cancer. J Immunother Cancer. (2024) 12(1):e007922. doi: 10.1136/jitc-2023-007922 38177076 PMC10773442

[12] Bytyci J Ying Y Lee LYW. Immunocompromised individuals are at increased risk of COVID-19 breakthrough infection, hospitalization, and death in the post-vaccination era: A systematic review. Immun Inflammation Dis. (2024) 12:e1259. doi: 10.1002/iid3.1259 PMC11044684 38661301

[13] Liang W Guan W Chen R Wang W Li J Xu K. Cancer patients in SARS-coV-2 infection: A nationwide analysis in China. Lancet Oncol. (2020) 21:335–7. doi: 10.1016/S1470-2045(20)30096-6 PMC7159000 32066541

[14] Alaeddini M Etemad-Moghadam S. SARS-COV-2 infection in cancer patients, susceptibility, outcome and care. Am J Med Sci. (2022) 364:511–20. doi: 10.1016/j.amjms.2022.05.017 PMC9119956 35605680

[15] Yin T Li Y Ying Y Luo Z. Prevalence of comorbidity in chinese patients with COVID-19: systematic review and meta-analysis of risk factors. BMC Infect Dis. (2021) 21:200. doi: 10.1186/s12879-021-05915-0 33618678 PMC7897883

[16] Carfì A Bernabei R Landi F. Persistent symptoms in patients after acute COVID-19. JAMA. (2020) 324:603–5. doi: 10.1001/jama.2020.12603 PMC7349096 32644129

[17] Seegers V Rousseau G Zhou K Blanc-Lapierre A Bigot F Mahammedi H. COVID-19 infection despite previous vaccination in cancer patients and healthcare workers: results from a french prospective multicenter cohort (Papesco-19). Cancers (Basel). (2023) 15(19):4777. doi: 10.3390/cancers15194777 37835471 PMC10571737

[18] Bell ML Catalfamo CJ Farland LV Ernst KC Jacobs ET Klimentidis YC. Post-acute sequelae of COVID-19 in a non-hospitalized cohort: results from the arizona covhort. PloS One. (2021) 16:e0254347. doi: 10.1371/journal.pone.0254347 34347785 PMC8336814

[19] Klein J Wood J Jaycox JR Dhodapkar RM Lu P Gehlhausen JR. Distinguishing features of long COVID identified through immune profiling. Nature. (2023) 623:139–48. doi: 10.1038/s41586-023-06651-y PMC10620090 37748514

[20] Brehm J Spaeth A Dreßler L Masetto T Dannenberg R Peter C. SARS-COV-2 antibody progression and neutralizing potential in mild symptomatic COVID-19 patients - a comparative long term post-infection study. Front Immunol. (2022) 13:915338. doi: 10.3389/fimmu.2022.915338 36059441 PMC9428854

[21] Cohen I Campisi-Pfinto S Rozenberg O Colodner R Bar-Sela G. The humoral response of patients with cancer to breakthrough COVID-19 infection or the fourth bnt162b2 vaccine dose. Oncologist. (2023) 28:e225–e7. doi: 10.1093/oncolo/oyad003 PMC10078898 36856804

[22] Zhao X Zhang R Qiao S Wang X Zhang W Ruan W. Omicron SARS-COV-2 neutralization from inactivated and zf2001 vaccines. N Engl J Med. (2022) 387:277–80. doi: 10.1056/NEJMc2206900 PMC9342420 35793198

[23] Peng W Ma X Tan K Wang H Cong M Zhang Y. Evaluation of cross-neutralizing antibodies in children infected with omicron sub-variants. Lancet Reg Health West Pac. (2023) 40:100939. doi: 10.1016/j.lanwpc.2023.100939 37953966 PMC10632766

[24] Zhang H Xu N Xu Y Qin P Dai R Xu B. Safety and immunogenicity of ad5-ncov immunization after three-dose priming with inactivated SARS-COV-2 vaccine in chinese adults. Nat Commun. (2023) 14:4757. doi: 10.1038/s41467-023-40489-2 37553338 PMC10409730

[25] Cervia-Hasler C Brüningk SC Hoch T Fan B Muzio G Thompson RC. Persistent complement dysregulation with signs of thromboinflammation in active long COVID. Science. (2024) 383:eadg7942. doi: 10.1126/science.adg7942 38236961

[26] Parsons RJ Acharya P. Evolution of the SARS-COV-2 omicron spike. Cell Rep. (2023) 42:113444. doi: 10.1016/j.celrep.2023.113444 37979169 PMC10782855

[27] Spiteri G D'Agostini M Abedini M Ditano G Collatuzzo G Boffetta P. Protective Role of SARS-COV-2 Anti-S Igg against Breakthrough Infections among European Healthcare Workers During Pre and Post-Omicron Surge-Orchestra Project. Infection. (2024) 52(4):1347–56. doi: 10.1007/s15010-024-02189-x PMC11289150 38326526

[28] Yang Y Guo L Yuan J Xu Z Gu Y Zhang J. Viral and antibody dynamics of acute infection with SARS-COV-2 omicron variant (B.1.1.529): A prospective cohort study from shenzhen, China. Lancet Microbe. (2023) 4:e632–e41. doi: 10.1016/s2666-5247(23)00139-8 37459867

[29] Regenhardt E Kirsten H Weiss M Lübbert C Stehr SN Remane Y. SARS-COV-2 vaccine breakthrough infections of omicron and delta variants in healthcare workers. Vaccines (Basel). (2023) 11(5):958. doi: 10.3390/vaccines11050958 37243062 PMC10220865

[30] Søraas A Grødeland G Granerud BK Ueland T Lind A Fevang B. Breakthrough infections with the omicron and delta variants of SARS-COV-2 result in similar re-activation of vaccine-induced immunity. Front Immunol. (2022) 13:964525. doi: 10.3389/fimmu.2022.964525 36159859 PMC9493489

[31] Prabani KIP Weerasekara I Damayanthi HDWT. COVID-19 vaccine acceptance and hesitancy among patients with cancer: A systematic review and meta-analysis. Public Health. (2022) 212:66–75. doi: 10.1016/j.puhe.2022.09.001 36244261 PMC9452406

[32] Poghosyan H Ni Z Vlahov D Nelson L Nam S. COVID-19 Vaccine Hesitancy among Medicare Beneficiaries with and without Cancer History: A Us Population-Based Study. J Community Health. (2023) 48:315–24. doi: 10.1007/s10900-022-01174-5 PMC9702715 36427111

[33] Qin S Li Y Wang L Zhao X Ma X Gao GF. Assessment of vaccinations and breakthrough infections after adjustment of the dynamic zero-COVID-19 strategy in China: an online survey. Emerg Microbes Infect. (2023) 12:2258232. doi: 10.1080/22221751.2023.2258232 37691586 PMC10512888

[34] Passaro A Peters S Mok TSK Attili I Mitsudomi T de Marinis F. Testing for COVID-19 in lung cancer patients. Ann Oncol. (2020) 31:832–4. doi: 10.1016/j.annonc.2020.04.002 PMC7144604 32278879

[35] Donze C Min V Ninove L de Lamballerie X Revon Rivière G Verschuur A. Bnt162b2 COVID-19 vaccines in children, adolescents and young adults with cancer-a 1-year follow-up. Vaccines. (2023) 11(5):989. doi: 10.3390/vaccines11050989 37243093 PMC10224057

[36] Guo L Wang G Wang Y Zhang Q Ren L Gu X. SARS-COV-2 -specific antibody and T-cell responses 1 year after infection in people recovered from COVID-19: A longitudinal cohort study. Lancet Microbe. (2022) 3:e348–e56. doi: 10.1016/S2666-5247(22)00036-2 PMC8942480 35345417

[37] Guo L Zhang Q Gu X Ren L Huang T Li Y. Durability and cross-reactive immune memory to SARS-COV-2 in individuals 2 years after recovery from COVID-19: A longitudinal cohort study. Lancet Microbe. (2024) 5:e24–33. doi: 10.1016/S2666-5247(23)00255-0 PMC10789611 38048805

[38] Li Y Qiao S Dong L Zhang R Li R Qin S. Antibody response assessment of immediate breakthrough infections after zero-COVID policy adjustment in China. Lancet Reg Health West Pac. (2023) 40:100945. doi: 10.1016/j.lanwpc.2023.100945 38033432 PMC10684796

[39] Hyun H Nham E Seong H Yoon JG Noh JY Cheong HJ. Long-Term Humoral and Cellular Immunity against Vaccine Strains and Omicron Subvariants (Bq.1.1, Bn.1, Xbb.1, and Eg.5) after Bivalent COVID-19 Vaccination. Front Immunol. (2024) 15:1385135. doi: 10.3389/fimmu.2024.1385135 38756783 PMC11096540

[40] Sil D Gautam S Saxena S Joshi S Kumar D Mehta A. Comprehensive analysis of omicron subvariants: eg.5 rise, vaccination strategies, and global impact. Curr Drug Targets. (2024) 25:517–25. doi: 10.2174/0113894501296586240430061915 38726782

[41] Soriano JB Murthy S Marshall JC Relan P Diaz JV. A clinical case definition of post-COVID-19 condition by a delphi consensus. Lancet Infect Dis. (2022) 22:e102–e7. doi: 10.1016/S1473-3099(21)00703-9 PMC8691845 34951953

[42] Ceban F Ling S Lui LMW Lee Y Gill H Teopiz KM. Fatigue and cognitive impairment in post-COVID-19 syndrome: A systematic review and meta-analysis. Brain Behav Immun. (2022) 101:93–135. doi: 10.1016/j.bbi.2021.12.020 PMC8715665 34973396

