# Supplementary material for: Clinical characteristics and antibody response to Omicron variants among solid carcinoma patients in China on the 2022.12–2023.4 wave of the COVID-19 pandemic
Source: Front Immunol. 2024 Nov 6;15:1476186. doi: 10.3389/fimmu.2024.1476186 (PMC11576375; doi:10.3389/fimmu.2024.1476186)
Supplement: Supplementary file 1 [file Table1.docx]

Supplementary Material

Clinical characteristics and antibody response to Omicron variants among solid carcinoma patients in China on the 2022.12–2023.4 wave of the COVID-19 pandemic

Dai Rongrong^1^^†^, Weiyu Peng^2†^, Nani Xu^3^, Pan Qin^3^, Linling Ding^4^, Qianhui Hua^4^, Jianmin Jiang^1,4*^, He Fang^5*^, Hangjie Zhang^4*^

# Supplementary Table

**Supplementary Table 1** Multifactor analysis of COVID-19 infection symptoms in solid carcinoma patients

|  | **COVID-19 infection** | | **General symptom** | | **Respiratory symptom** | | **Digestive tract symptom** | | | **Fever(≥38°C)** | | **Muscular soreness** | |
| --- | --- | --- | --- | --- | --- | --- | --- | --- | --- | --- | --- | --- | --- |
|  | **OR(95%CI)** | **p** | **OR(95%CI)** | **p** | **OR(95%CI)** | **p** | **OR(95%CI)** | **p** | **OR(95%CI)** | | **p** | **OR(95%CI)** | **p** |
| **Gender** |  |  |  |  |  |  |  |  |  | |  |  |  |
| Female | ref | / | ref | / | ref | / | ref | / | ref | | / | ref | / |
| Male | 0.3(0.0,3.3) | 0.343 | / | / | 0.6(0.1,3.6) | 0.590 | 1.1(0.1,20.1) | 0.949 | 4.5(0.3,78.2) | | 0.301 | 1.3(0.2,8.4) | 0.770 |
| **Age** |  |  |  |  |  |  |  |  |  | |  |  |  |
| 18-59 years | ref | / | ref | / | ref | / | ref | / | ref | | / | ref | / |
| ≥60 years | 2.9(0.4,18.6) | 0.267 | 0.2(0.0,3.2) | 0.239 | 0.9(0.2,3.6) | 0.845 | 1.7(0.2,15.3) | 0.633 | 0.3(0.1,2.2) | | 0.264 | 1.9(0.4,8.6) | 0.397 |
| **BMI** |  |  |  |  |  |  |  |  |  | |  |  |  |
| <25.0 kg/m^2^ | ref | / | ref | / | ref | / | ref | / | ref | | / | ref | / |
| ≥25.0 kg/m^2^ | 0.3(0.0,1.7) | 0.163 | 0.3(0.0,4.2) | 0.353 | 0.5(0.1,2.4) | 0.387 | / | / | 0.6(0.1,3.8) | | 0.619 | 1.6(0.3,7.7) | 0.573 |
| **Vaccine(s) administered** | | | |  |  |  |  |  |  | |  |  |  |
| No | ref | / | ref | / | ref | / | ref | / | ref | | / | ref | / |
| Yes | 91.5(2.0,4086.5) | 0.020 | / | / | 0.6(0.0,20.0) | 0.763 | / | / | / | | / | 1.2(0.0,42.5) | 0.902 |
| **Type of malignancy** | |  |  |  |  |  |  |  |  | |  |  |  |
| Lung carcinoma | ref | / | ref | / | ref | / | ref | / | ref | | / | ref | / |
| Digestive carcinoma | 1.6(0.1,35.9) | 0.778 | / | / | 0.3  (0.0,4.9) | 0.426 | / | / | 1.3(0.0,35.1) | | 0.873 | 4.7(0.3,80.2) | 0.282 |
| Liver carcinoma | / | / | / | / | / | / | / | / | / | | / | / | / |
| Breast carcinoma | 0.3(0.0,6.0) | 0.437 | 6.6(0.2,252.8) | 6.643 | 1.3(0.1,16.8) | 0.866 | / | / | 1.2(0.1,24.0) | | 0.906 | 9.3(0.5,167.9) | 0.132 |
| Thyroid carcinoma | 0.6(0.0,16.7) | 0.748 | / | / | 0.9(0.0,16.4) | 0.939 | / | / | 1.3(0.0,43.6) | | 0.868 | 23.8(0.8,750.5) | 0.072 |
| Prostate carcinoma | / | / | / | / | 0.5(0.0,7.5) | 0.611 | / | / | 0.2(0.0,6.4) | | 0.400 | 0.9(0.1,17.3) | 0.964 |
| Other carcinoma | 1.0(0.1,14.9) | 0.996 | 9.0(0.2,376.1) | 8.982 | 0.9(0.1,8.8) | 0.903 | / | / | 4.6(0.2,109.2) | | 0.347 | 2.0(0.2,27.4) | 0.595 |

Data are OR(95%CI). Statistical analysis was conducted using the Binary logistic regression

**Supplementary Table 2 Multiple linear regression analysis of antibody levels in solid carcinoma patients**

|  | **Anti-RBD IgG** | | **BA.4/5** | | **BF.7** | |
| --- | --- | --- | --- | --- | --- | --- |
|  | **B** | **p** | **B** | **p** | **B** | **p** |
| **Gender** | | | | | | |
| Female | ref | / | ref | / | ref | / |
| Male | -0.2(-0.5,0.2) | 0.364 | 0.7(-0.4,1.8) | 0.209 | -0.7(-1.9,0.4) | 0.219 |
| **Age** | | | | | | |
| 18-59 years | ref | / | ref | / | ref | / |
| ≥60 years | 0.3(-0.1,0.7) | 0.163 | -0.7(-1.9,0.4) | 0.219 | 0.1(-1.1,1.3) | 0.865 |
| **BMI** | | | | | | |
| <25.0 | ref | / | ref | / | ref | / |
| ≥25.0 | 0.1(-0.3,0.5) | 0.688 | -0.4(-1.6,0.8) | 0.512 | 0.2(-1.1,1.4) | 0.812 |
| **Vaccine(s) administered** | | | | | | |
| No | ref | / | ref | / | ref | / |
| Yes | 0.5 (-0.6,1.6) | 0.357 | -2.9(-6.0,0.2) | 0.066 | -3.0(-6.2,0.2) | 0.064 |
| **COVID-19 infection** | | | | | | |
| No | ref | / | ref | / | ref | / |
| Yes | 1.4(0.8,2.0) | <0.001 | 2.4(0.7,4.1) | 0.007 | 5.3(3.5,7.1) | <0.001 |
| **Type of malignancy** | | | | | | |
|  | 0.0(-0.1,0.1) | 0.676 | 0.0(-0.2,0.3) | 0.947 | 0.1(-0.2,0.34) | 0.526 |

Data are B (95%CI). Statistical analysis was conducted using the linear regression analysis. Type of malignancy:1-Lung cancer;;2-Digestive cancer;3-Liver cancer;4-Breast cancer;5-Thyroid cancer;6-Prostate cancer;7-Other cancers.

**
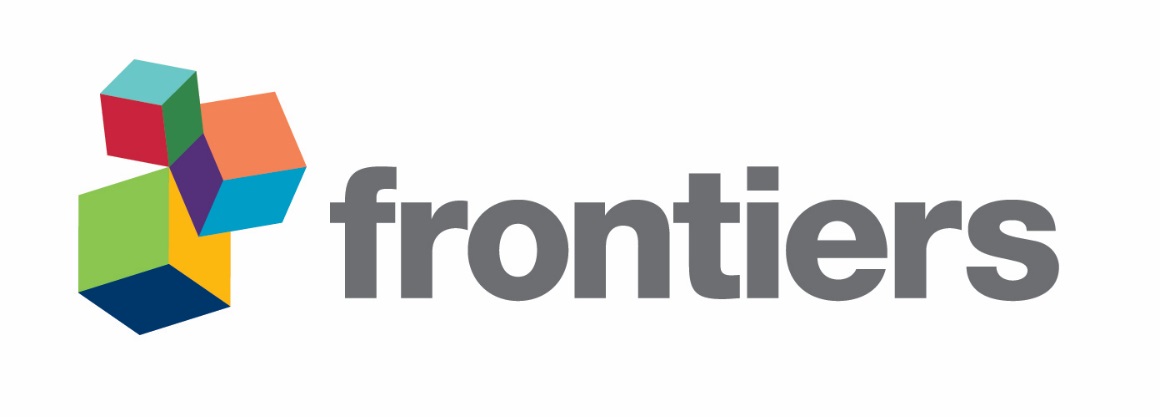
**
